# Bilateral Revision for Periprosthetic Osteolysis Around Primary Total Knee Prostheses: A Case Report

**DOI:** 10.7759/cureus.113088

**Published:** 2026-07-21

**Authors:** Laury Angenent, Robin Van Kempen

**Affiliations:** 1 Department of Orthopedics and Traumatology, Catharina Hospital, Eindhoven, NLD

**Keywords:** complex arthroplasty, orthopedic surgery, periprosthetic osteolysis, revision arthroplasty, total knee arthroplasty

## Abstract

A 73-year-old female with a history of thrombosis and anemia presented with bilateral knee pain and an inability to walk. Clinical examination and radiographs showed extensive bilateral periprosthetic osteolysis (PPOL) without signs of inflammation. She successfully underwent bilateral revision arthroplasty with ligament reconstruction. PPOL is a multifactorial process in which radiologic findings may not correlate with the clinical presentation. This case supports the discussion of whether long-term follow-up is sufficient or earlier intervention is warranted.

## Introduction

Total knee arthroplasty (TKA) is a successful surgical procedure for end-stage osteoarthritis. Despite high success rates, the risk of revision ranges from 3.6% to 15.4%, depending on patient- and implant-related factors [1]. One cause of failure is periprosthetic osteolysis (PPOL), often triggered by wear-induced particles from implant components (e.g., polyethylene (PE) and metal debris), which induce chronic inflammation, stimulate osteoclastic activity, and suppress bone formation. The process is considered multifactorial, involving surgeon-, patient-, and implant-related factors, and has been extensively reviewed by Gallo et al. in 2013 [2].

PPOL may remain clinically silent for a prolonged period. This has been attributed to the slowly progressive nature of osteoclast-mediated bone resorption, the minimal nociceptive stimulation associated with early periprosthetic osteolytic lesions, and the fact that symptoms usually develop only after mechanical complications, such as cortical bone involvement, implant loosening, or instability, have occurred.

Case reports describe PPOL due to PE wear [3], galvanic corrosion [4], metallosis [5], mechanical stress [6], inflammatory responses [7], or a combination of these factors. We review the etiology of PPOL and present a unique case of bilateral TKA with extensive tibial osteolysis that remained asymptomatic for a prolonged period. This case highlights the need for radiologic follow-up, even in the absence of symptoms, to detect progressive osteolysis early. We describe the diagnosis, treatment using the Megasystem-C^®^ modular prosthesis system (Waldemar Link GmbH & Co. KG, Hamburg, Germany), and follow-up.

## Case presentation

First presentation

**Figure 1:**
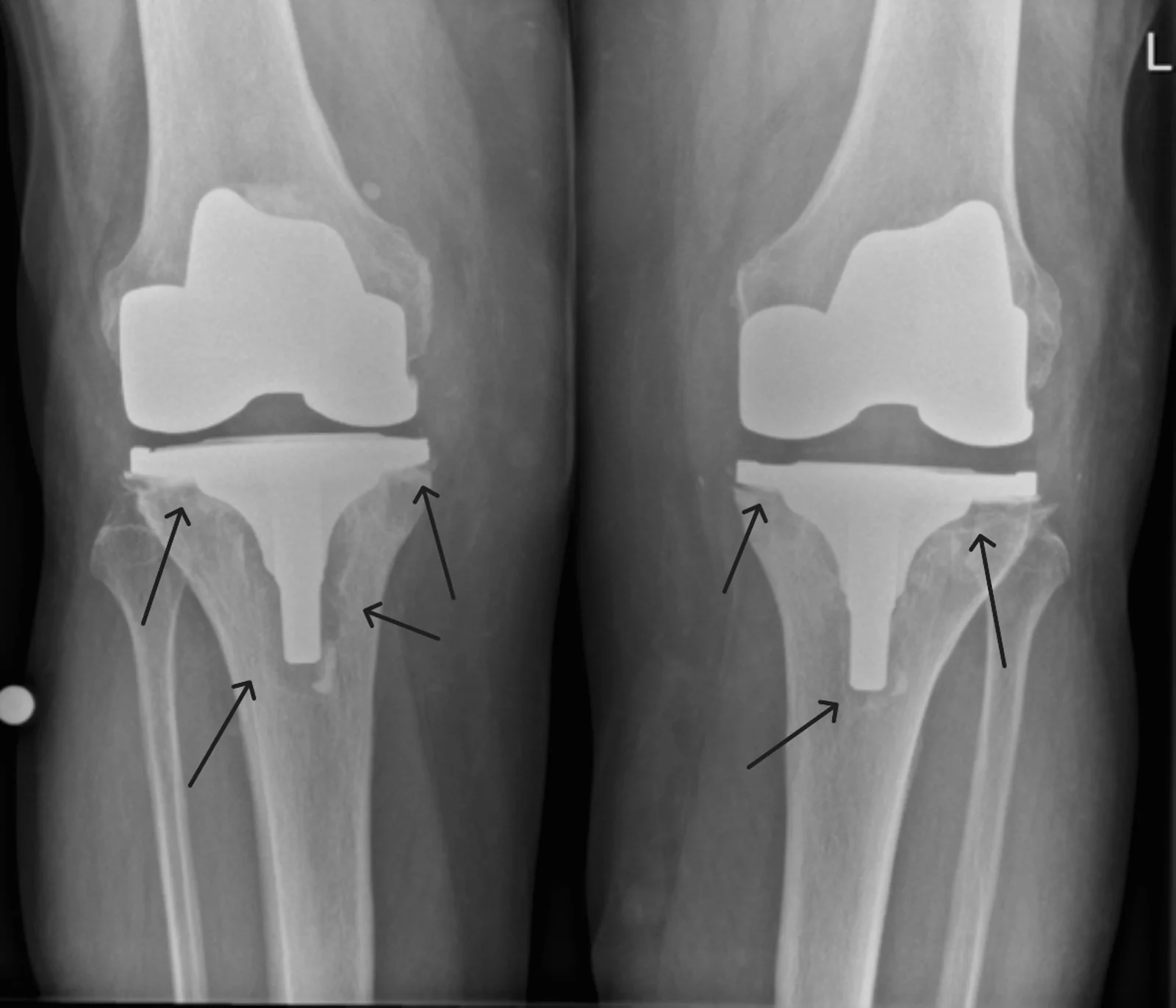
Anteroposterior radiograph of both knees showing minimal signs of osteolysis surrounding the TKAs five years after primary TKA. TKA, total knee arthroplasty

**Figure 2:**
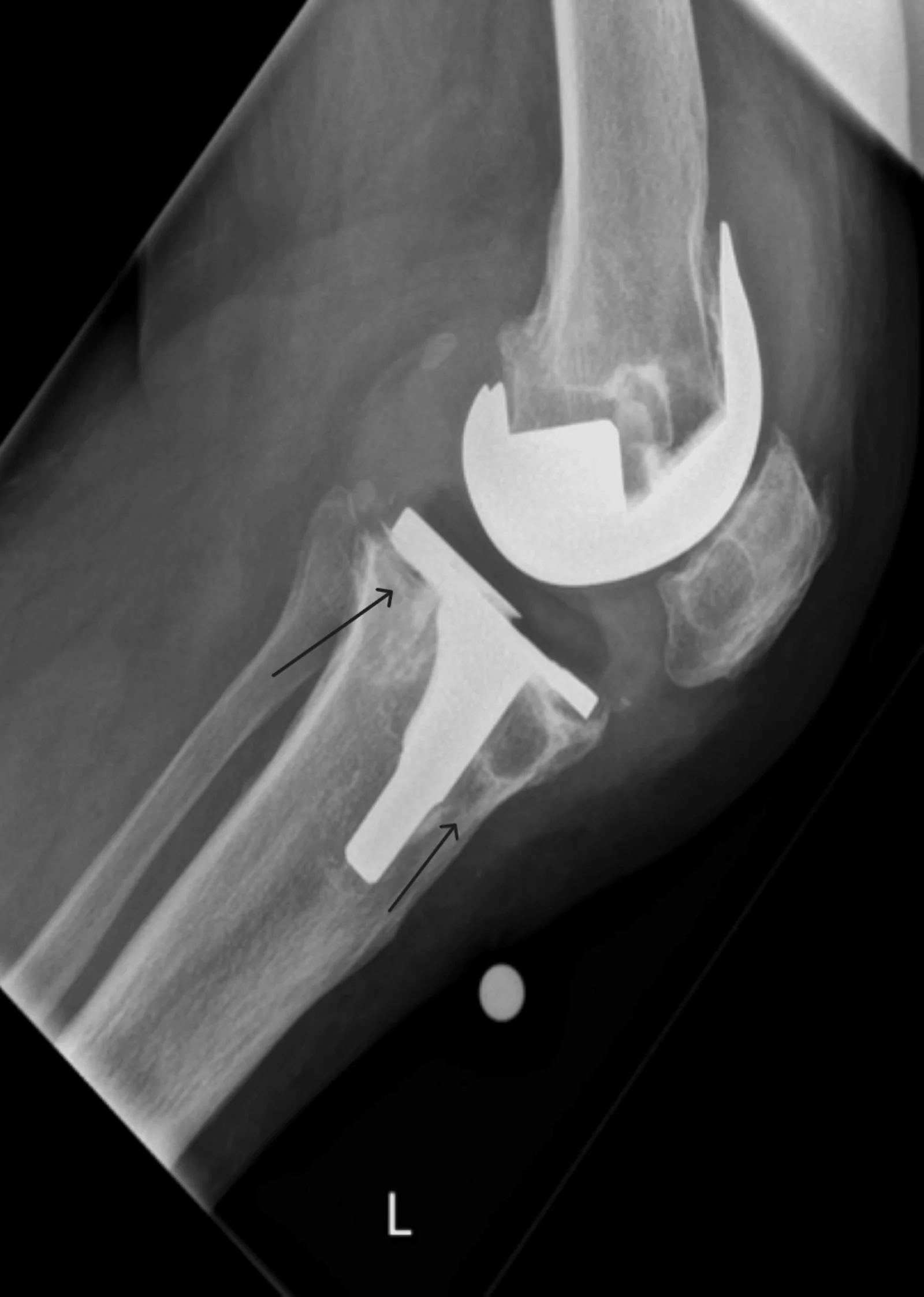
Lateral radiograph of the left knee showing minimal signs of osteolysis surrounding the TKA five years after primary TKA. TKA, total knee arthroplasty

**Figure 3:**
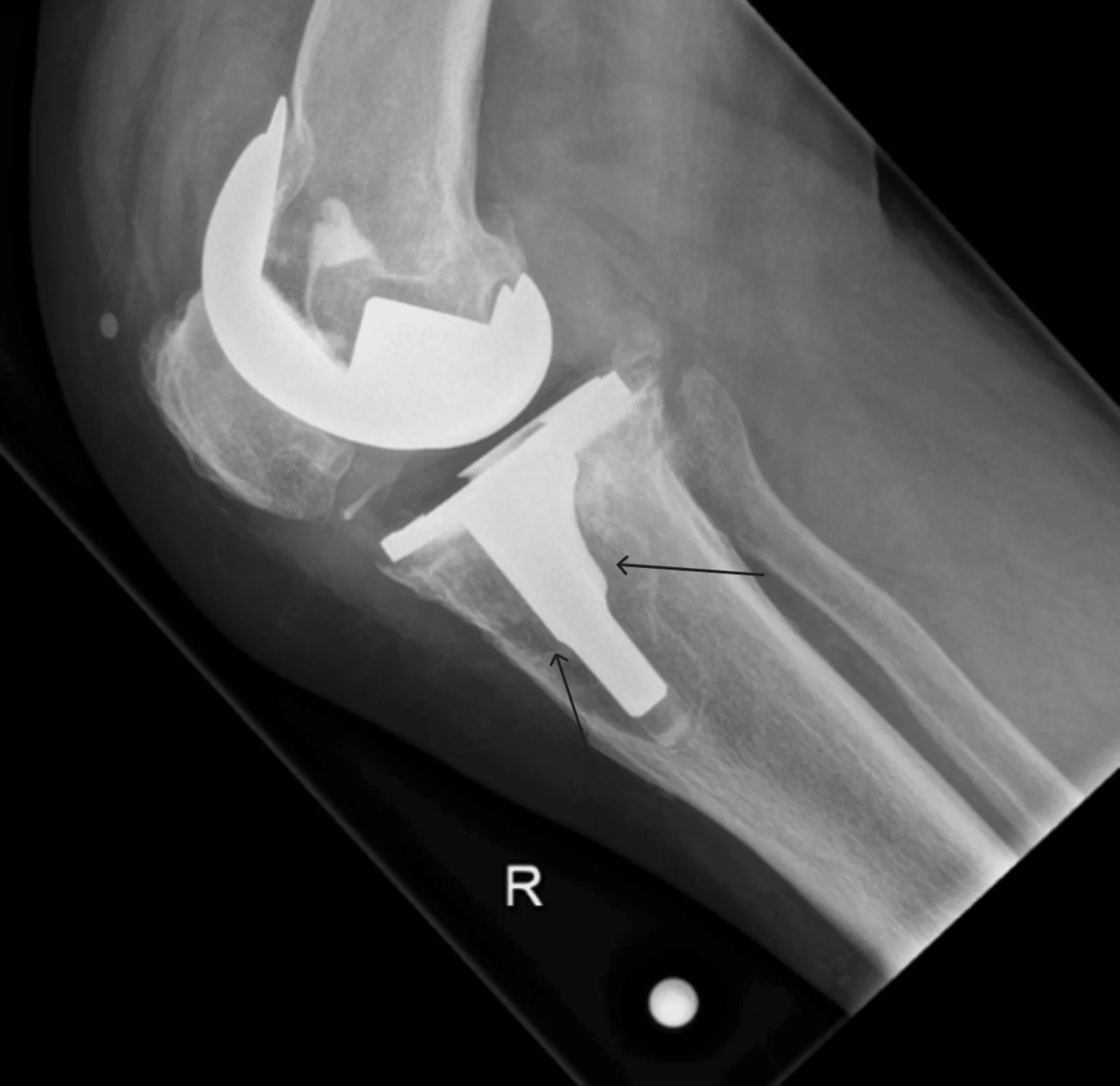
Lateral radiograph of the right knee showing minimal signs of osteolysis surrounding the TKA five years after primary TKA. TKA, total knee arthroplasty

A 73-year-old woman with a medical history of thrombosis and anemia was referred to our Department of Orthopedic Surgery in 2024 with progressive complaints involving both knees and legs. In 2015, a cemented TKA without patellar resurfacing was implanted in the right knee, followed by implantation in the left knee in 2016, both for debilitating osteoarthritis. There were no issues with rehabilitation or wound healing at that time. However, the patient experienced mild discomfort in the back of both knees. Regular follow-up was conducted for five years. She reported no limitations in activities of daily living, although her walking distance remained limited. Her last visit to an orthopedic surgeon was six years ago. Radiographs obtained at that time showed lucency (Figures 1-3). However, the patient was asymptomatic; therefore, no further follow-up was planned. Over the subsequent two years, she developed progressively worsening symptoms in both knees and presented to our institution with near inability to walk and continuous pain. To maintain some level of fitness, the patient cycled for about 30 minutes per day on an exercise bike.

Upon physical examination, no abnormal soft tissue findings were observed. The patient was able to fully extend both legs. Marked instability was noted, and the tip of the tibial stem component was palpable at the level of the tibial tuberosity bilaterally. Blood test results were normal (Table 1).

**Table 1:** Laboratory findings at presentation.

Parameter	Patient value	Reference range
Erythrocyte sedimentation rate	31 mm/hour	<30 mm/hour
Leukocytes	8.0 × 10⁹/L	4-10 × 10⁹/L
C-reactive protein	<6.0 mg/L	<6.0 mg/L

**Figure 4:**
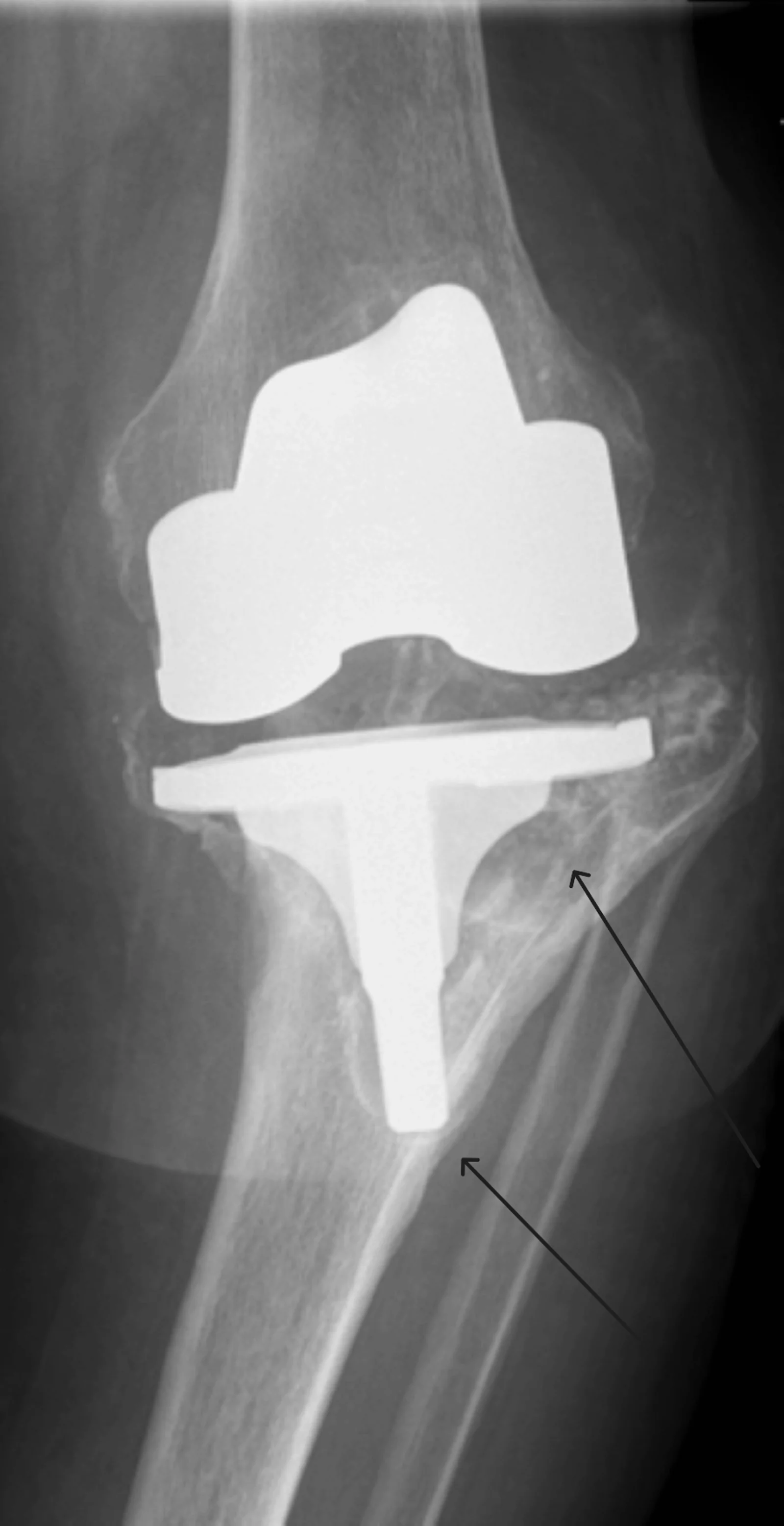
Anteroposterior radiograph of the left knee showing osteolysis of the proximal tibia approximately nine years after primary TKA. TKA, total knee arthroplasty

**Figure 5:**
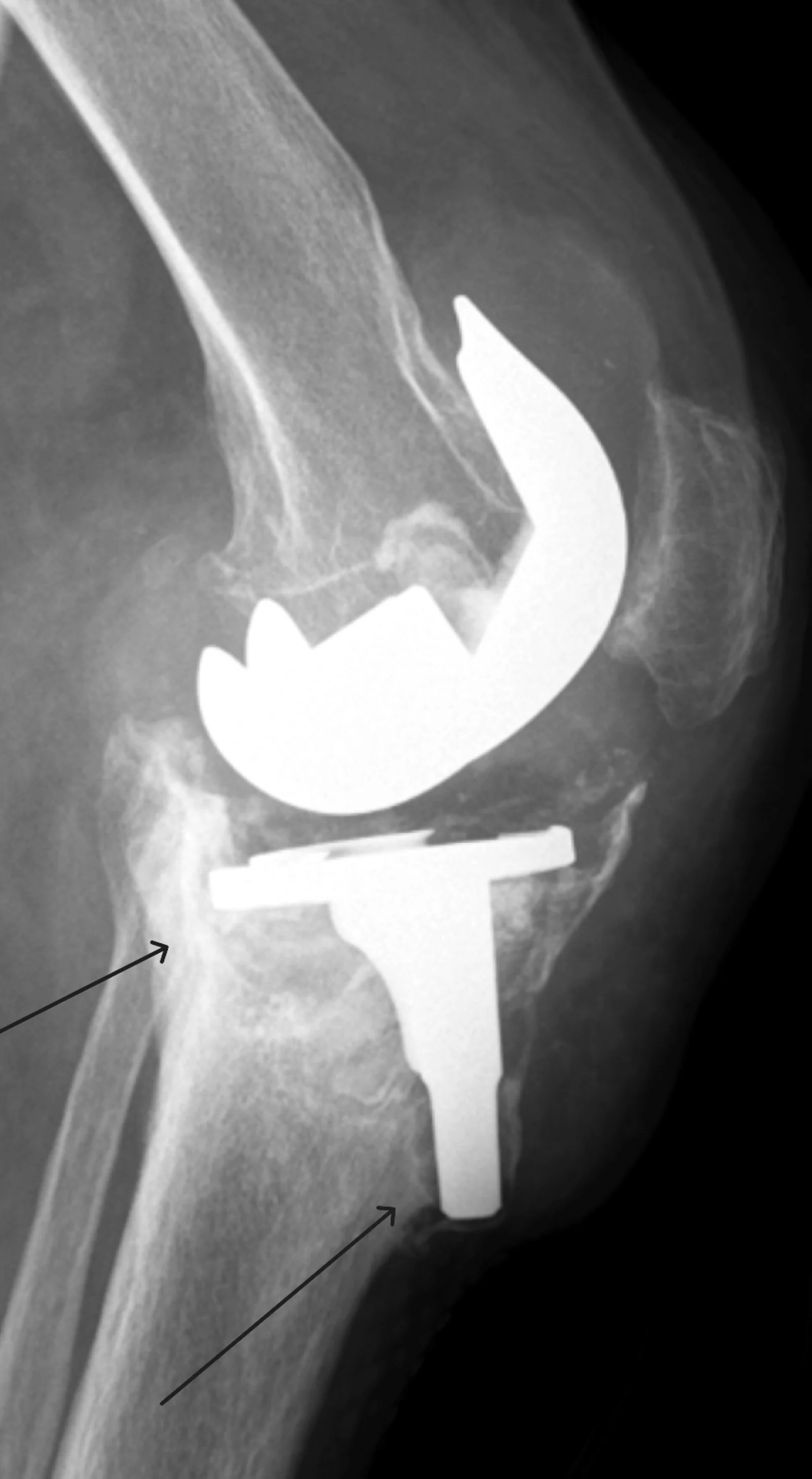
Lateral radiograph of the left knee showing osteolysis of the proximal tibia approximately nine years after primary TKA. TKA, total knee arthroplasty

**Figure 6:**
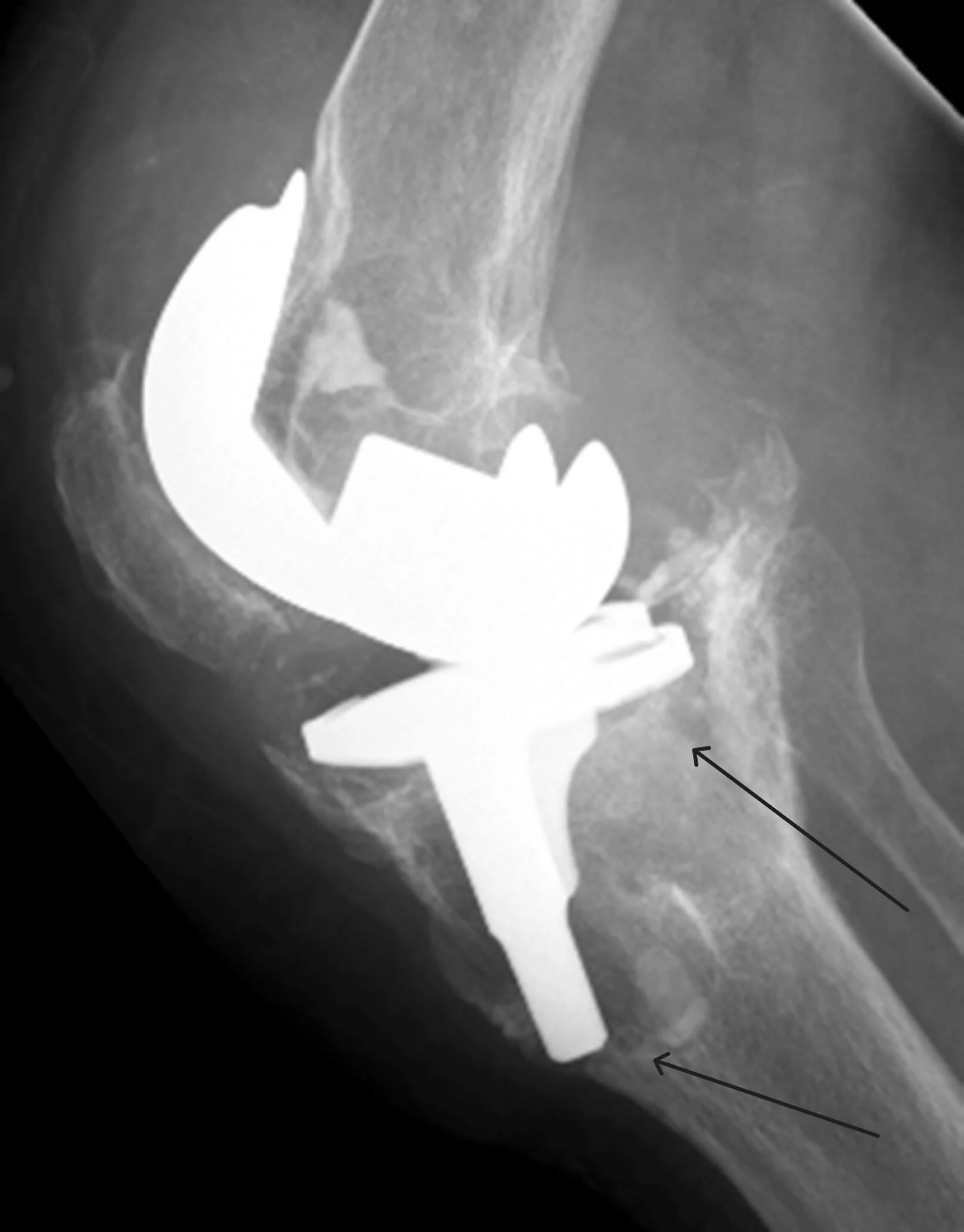
Lateral radiograph of the right knee showing osteolysis of the proximal tibia approximately nine years after primary TKA. TKA, total knee arthroplasty

**Figure 7:**
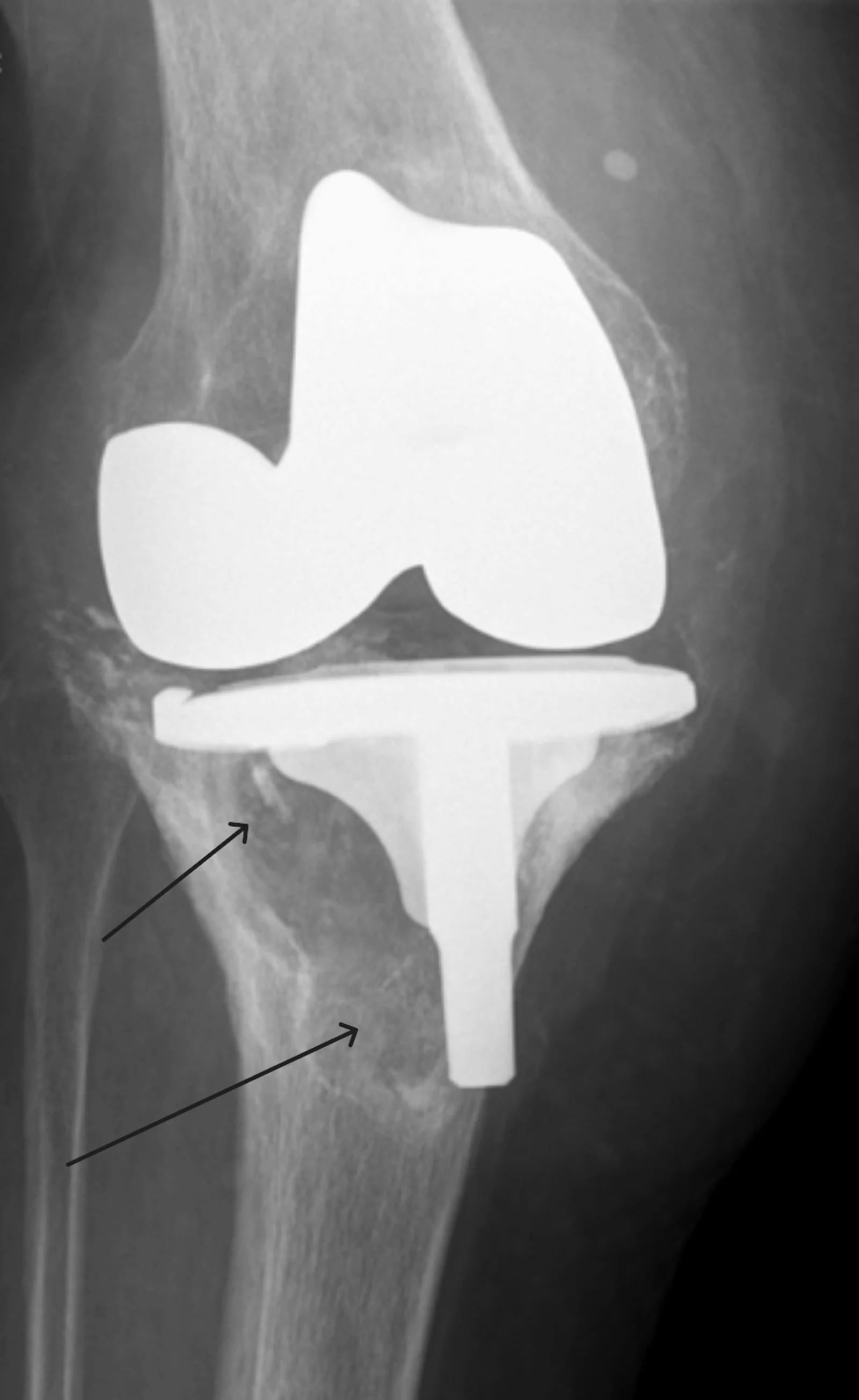
Anteroposterior radiograph of the right knee showing osteolysis of the proximal tibia approximately nine years after primary TKA. TKA, total knee arthroplasty

Radiographs showed massive tibial and femoral PPOL in both knees, classified as F2B/T3 according to the Anderson Orthopaedic Research Institute classification (Figures 4-7) [8]. Computed tomography demonstrated massive osteolysis around the tibial component in the right knee. Multiple cortical defects were seen on the ventral, dorsal, and lateral sides. The tibial stem had broken through the ventral cortex, and minimal lucency was observed at the notch of the femoral component. The left knee showed extensive osteolysis around the tibial component, with multiple cortical defects on the medial, dorsal, and ventral sides. No lucency was seen around the femoral component.

To rule out a low-grade infection, intra-articular aspiration of both knees was performed. Normal leukocyte count and differential were found. The synovial fluid alpha-defensin test (Synovasure^®^, Zimmer Biomet, Inc., Warsaw, IN, USA) and microbiological cultures were negative for both knees. A diagnosis of aseptic loosening of the tibial component in both knees was made, and a one-stage revision involving tibial replacement with augmentation of the patellar tendon using a Ligament Augmentation and Reconstruction System (LARS) was planned.

Surgical procedure

An experienced revision TKA (rTKA) surgeon performed bilateral staged revision arthroplasties. The left knee was revised first, revealing femoral component loosening and tibial migration without PE wear. Bone defects were classified as F2A/T3. A Megasystem-C^®^ prosthesis was implanted with tibial tuberosity fixation using LARS and FiberWire^®^ sutures. Intraoperatively, flexion reached 110° with full extension. Postoperative care included five days of cefazolin, five weeks of dalteparin, 48 hours of bed rest followed by progressive weight-bearing (up to 50%) with a hinged brace, and passive flexion up to 60°. Intraoperative cultures were negative, and postoperative radiographs confirmed correct implant positioning.

Two weeks later, rTKA of the right knee was performed with similar findings, including femoral component loosening and tibial migration without PE wear. Bone defects were classified as F3/T4. A Megasystem-C^®^ prosthesis was implanted using identical fixation techniques and the same postoperative protocol. After 48 hours, extension training began for the right leg, while weight-bearing on the left leg was increased under physiotherapy guidance.

Follow-up

After discharge, both knees were braced (left, 0°-60°; right, 0°-45°), with limited mobilization (maximum 50% weight-bearing) for seven weeks. Radiographs obtained at week 7 showed callus formation in the left femur and a protruding screw near the patellar tendon, which was surgically stabilized. The same complication occurred in the right knee three weeks later, requiring similar refixation. No wound complications were observed in either knee.

**Figure 8:**
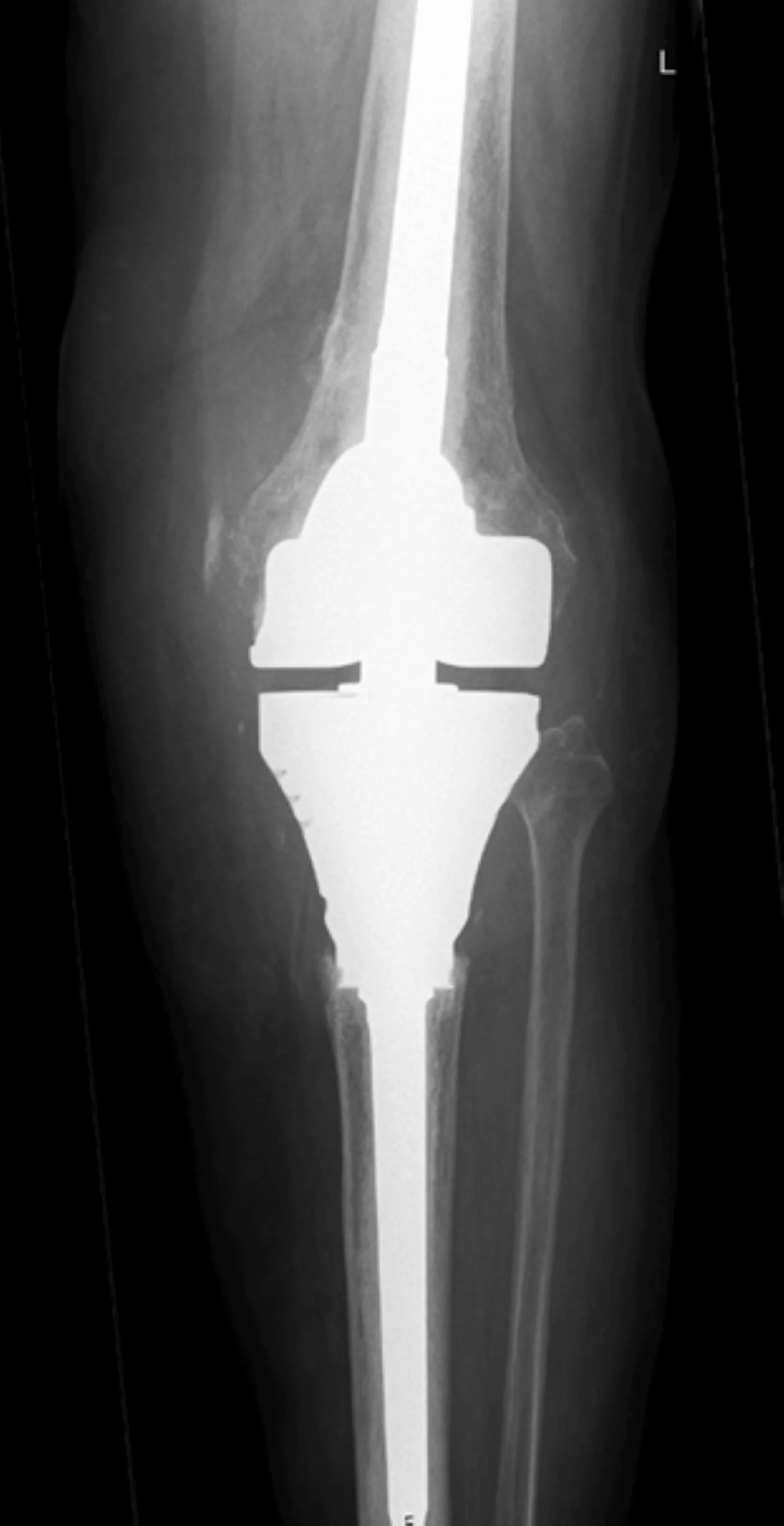
Anteroposterior radiograph of the left knee one year postoperatively showing appropriate implant positioning and no complications after revision arthroplasty.

**Figure 9:**
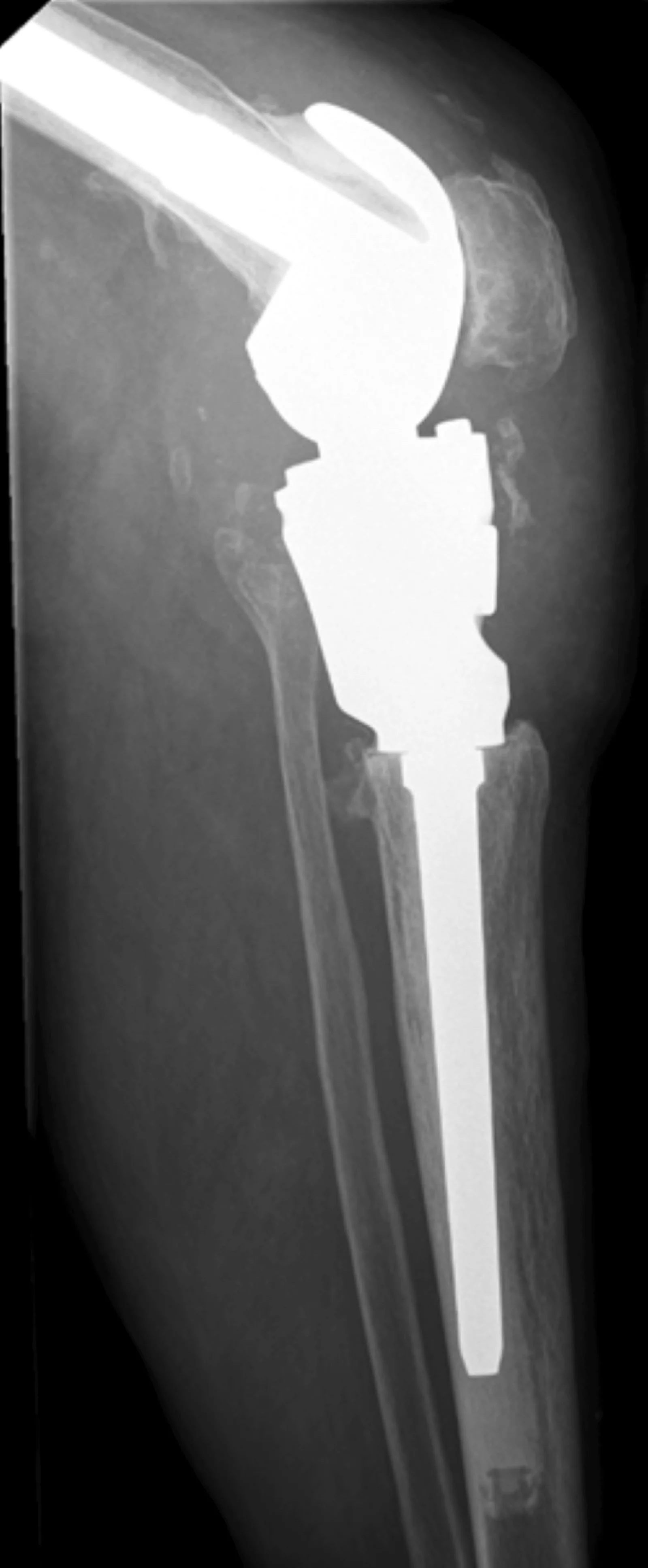
Lateral radiograph of the left knee one year postoperatively showing appropriate implant positioning and no complications after revision arthroplasty.

**Figure 10:**
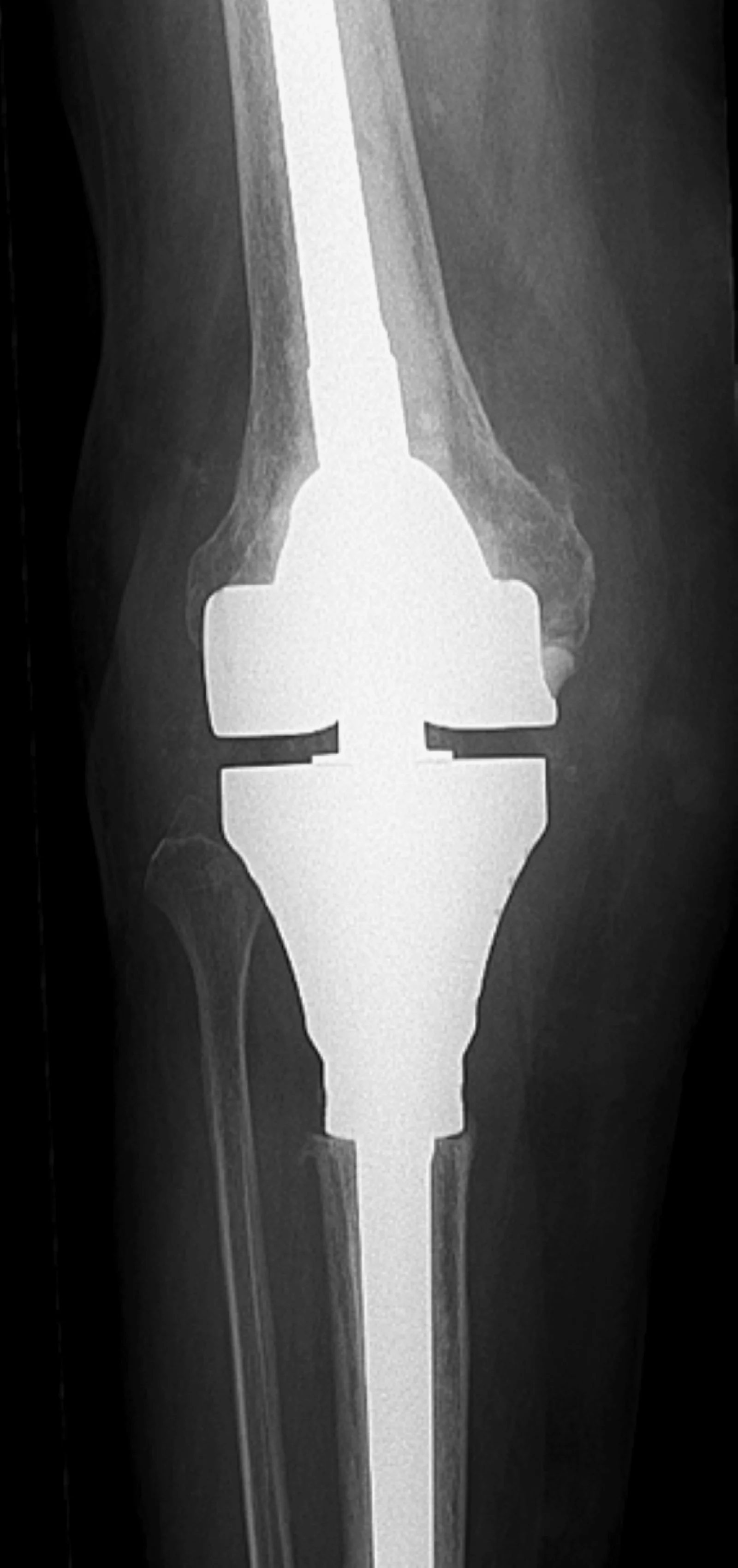
Anteroposterior radiograph of the right knee one year postoperatively showing appropriate implant positioning and no complications after revision arthroplasty.

**Figure 11:**
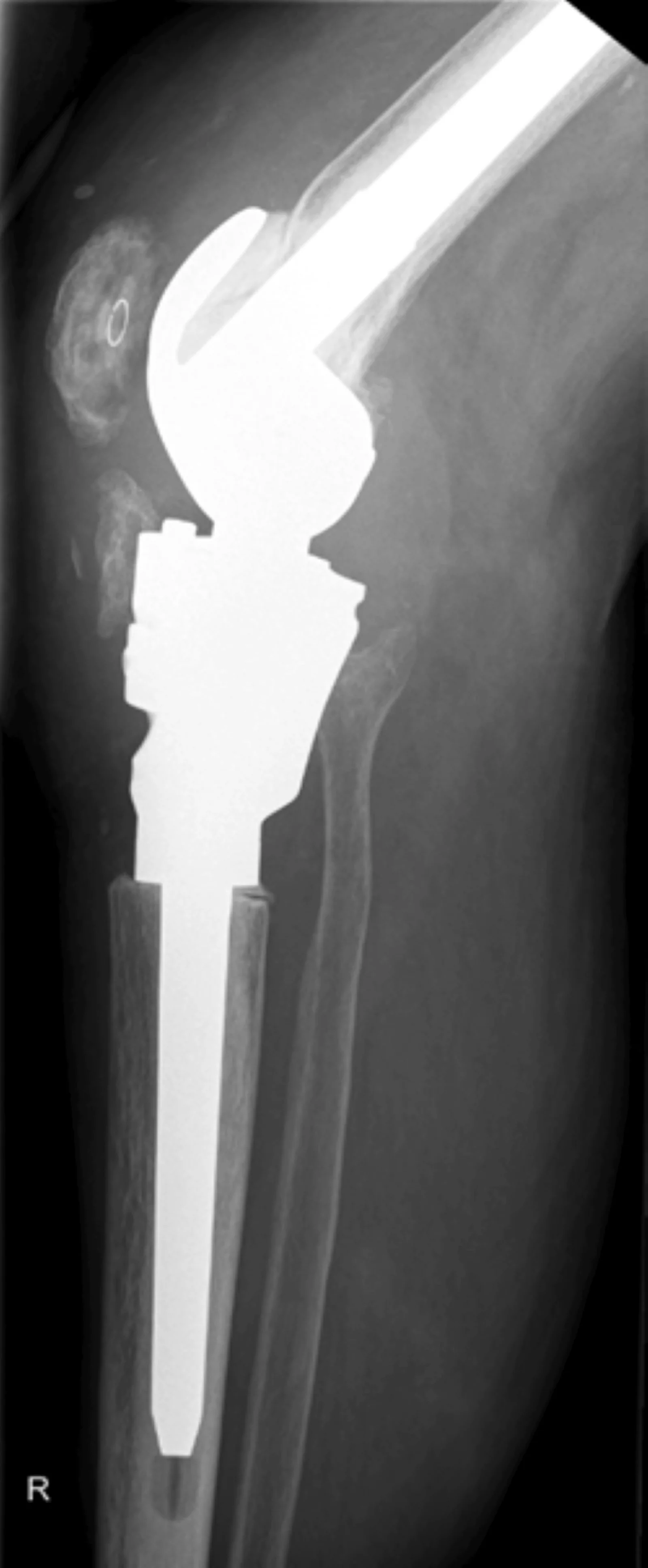
Lateral radiograph of the right knee one year postoperatively showing appropriate implant positioning and no complications after revision arthroplasty.

Four months postoperatively, the patient had flexion of 70° in the left knee and 80° in the right knee. After six months, both knees had full extension, with flexion of 80° in the left knee and 90° in the right knee, accompanied by minimal pain and improved mobility. At the one-year follow-up, she walked independently indoors, used one crutch outdoors, achieved a walking distance of 1 km, and rode a tricycle. One-year postoperative radiographs showed stable fixation and no complications (Figures 8-11). The patient was very satisfied with the outcome.

## Discussion

The survival rate of TKA is influenced by multiple factors, one of which is early bone remodeling. Loss of bone mineral density is a normal physiological process and occurs mostly within the first postoperative year, stabilizing thereafter [9]. Pathological mechanical forces acting on the prosthesis surface can lead to degradation of the bone-cement interface, which is crucial for implant fixation and long-term survival and can therefore contribute to PPOL. Abnormal loading conditions are often associated with malalignment and PPOL [10].

PE is another significant contributor to PPOL. Wear particles, combined with joint movement, stimulate the formation of a pseudosynovial membrane that secretes fluid [11]. Fluid accumulation within the joint cavity generates hydrodynamic forces affecting the periprosthetic joint space during knee motion. Intermittent pressure waves act upon the bone bed, soft tissues, and bone-implant interface, contributing to osteolytic processes [2,12]. In response to phagocytosis and nonphagocytic stimulation, macrophages and other immune cells express chemokines, cytokines, growth factors, and other inflammatory mediators. This immune response leads to an increased presence and activation of osteoclasts at the bone-implant interface, promoting periprosthetic bone resorption. Inflammatory signaling pathways facilitate expansion of pseudosynovial and granulomatous tissues while promoting fluid secretion, all of which contribute to the expansion of resorption cavities around the prosthesis [13,14].

Wear debris stimulates an innate immune response, causing chronic low-grade inflammation and increased osteoclast formation, accumulation, and maturation [15]. The osteoblast phenotype in periprosthetic bone may undergo alterations favoring osteoclast differentiation while inhibiting new bone formation, possibly leading to loss of bone mass [16]. The receptor activator of nuclear factor kappa-B ligand (RANKL)-receptor activator of nuclear factor kappa-B-osteoprotegerin (OPG) interaction plays an essential role in osteoclastogenesis at the bone surface. Tumor necrosis factor-α contributes to the survival and differentiation of osteoclasts, whereas interleukin-1β plays a role in osteoclast activation [17]. Major sources of RANKL within the periprosthetic membrane include osteoblasts, bone marrow stromal cells, fibroblasts, and lymphocytes [18]. Previous studies found elevated RANKL levels in the interface membranes of patients undergoing revision surgery due to osteolysis [19]. Osteoclast maturation is inhibited by OPG, thereby decreasing bone resorption. The local ratio of RANKL to OPG may correlate with the size of the osteolytic defect [20].

Another factor contributing to osteolysis is the presence of voids at the cement-bone interface. These are inevitable after TKA implantation. The voids are filled with fibrous tissue containing few cells and blood vessels. Repeated mechanical stress induces trauma and hypoxic conditions, leading to fibroblast proliferation and extracellular matrix synthesis to withstand stresses around the prosthesis [2].

The clinical message of this case is the potential discordance between clinical presentation and structural deterioration. We presented a patient who was still able to exercise on a stationary bicycle while radiographs showed extensive bilateral PPOL. Single-stage rTKA was performed on both knees using Megasystem-C^®^ implants and LARS. During revision surgery, synovectomy, PE exchange, component revision, and ligament reconstruction were performed. There were no signs of advanced PE wear or damage to the metal components. At the one-year follow-up after revision, the patient remained asymptomatic, with no new osteolytic lesions on radiographs, and continued to enjoy a good quality of life and knee function.

Our case raises several important considerations regarding the pathogenesis and clinical presentation of PPOL. Despite the extensive osteolysis observed on radiographs, the patient experienced only mild symptoms before rTKA. The discrepancy between radiologic findings and the clinical presentation suggests that osteolysis may progress silently, with symptoms becoming apparent only in advanced stages when mechanical instability occurs.

Reflecting on the described mechanisms of implant loosening, various factors could have contributed to the osteolysis in our case. Since there were no signs of significant PE wear, metallosis, or infection, the osteolysis may have resulted from voids at the bone-cement interface or patient-specific bone remodeling dynamics. This case underscores the importance of adequate long-term radiographic surveillance in asymptomatic patients. Radiographs obtained at the five-year follow-up showed signs of osteolysis, yet the patient experienced only mild symptoms without pain or limitations in daily activities. It is also possible that the clinical manifestations of PPOL lagged behind the radiographic progression at that time. This case contributes to the debate on whether long-term follow-up is sufficient or whether earlier intervention is warranted in asymptomatic patients with radiographic abnormalities.

## Conclusions

PPOL after TKA is a multifactorial process in which mechanical stress, biological responses to wear particles, and changes at the bone-cement interface collectively lead to bone resorption and implant loosening. Clinical symptoms may lag behind radiographic findings, allowing osteolysis to progress silently. This case highlights the importance of long-term radiographic follow-up, even in asymptomatic patients, and contributes to the discussion of whether earlier intervention may be warranted.
